# Predictive Value of Nutrition Indices in Advanced Hepatocellular Carcinoma Patients Treated With Lenvatinib

**DOI:** 10.1002/kjm2.70230

**Published:** 2026-05-07

**Authors:** Cheng‐Wei Kuo, Wei‐Chen Tai, Yen‐Hao Chen, Ming‐Chao Tsai, Jing‐Houng Wang, Sheng‐Nan Lu, Tsung‐Hui Hu, Chao‐Hung Hung, Chien‐Hung Chen, Yuan‐Hung Kuo

**Affiliations:** ^1^ Divison of Hepatogastroenterology Kaohsiung Chang Gung Memorial Hospital Kaohsiung City Taiwan; ^2^ Division of Oncology Kaohsiung Chang Gung Memorial Hospital Kaohsiung City Taiwan

**Keywords:** geriatric nutritional risk index, hepatocellular carcinoma, lenvatinib, prognostic nutritional index

## Abstract

The prognostic nutritional index (PNI) and geriatric nutritional risk index (GNRI) are simple markers that reflect immune and nutritional status. This retrospective study evaluated their prognostic significance in 276 patients with advanced hepatocellular carcinoma (HCC) who received first‐line lenvatinib. PNI and GNRI were calculated based on serum albumin, lymphocyte count, and body weight. Patients were classified into high and low groups for each index. Multivariate models were used to assess their prognostic value for progression‐free survival (PFS) and overall survival (OS). Higher PNI (cut‐off: 45) and GNRI (cut‐off: 98) scores were associated with significantly better outcomes: the high PNI and low PNI groups achieved median PFS of 7.5 vs. 4.0 months (*p* = 0.002) and OS of 19.3 vs. 9.8 months (*p* < 0.001), respectively. Similarly, the high GNRI and low GNRI groups achieved median PFS of 7.3 versus 4.7 months (*p* = 0.001) and OS of 21 versus 9.8 months (*p* < 0.001), respectively. Patients with higher indices also had higher objective response rates, lower rates of severe treatment‐related adverse events, and were more likely to receive posttreatment therapies. The PNI (hazard ratio [HR]: 0.678, *p* = 0.018), GNRI (HR: 0.679, *p* = 0.031), and the combined indices (HR: 0.655, *p* = 0.013) independently demonstrated prognostic value for mortality in multivariate models. PNI and GNRI, both individually and in combination, are simple yet powerful tools that predict survival and treatment tolerance in patients with HCC receiving lenvatinib. Routine assessment of these indices may enhance patient stratification and guide therapeutic strategies.

**Abbreviations**
BCLC: Barcelona Clinic Liver Cancer
GNRI: geriatric nutritional risk index
HCC: hepatocellular carcinoma
OS: overall survival
PFS: progression‐free survival
PNI: prognostic nutritional index
TRAE: treatment‐related adverse event

## 1 Introduction

Hepatocellular carcinoma (HCC) is the third leading cause of cancer‐related mortality worldwide, accounting for approximately 800,000 deaths annually [1]. Early‐stage HCC can be treated with curative locoregional therapies; however, most symptomatic patients are diagnosed at an advanced stage and face a poor prognosis, as there is a limited range of effective treatment options. In recent years, the development of systemic therapies—including targeted therapies and immunotherapies—has expanded the treatment options and improved the survival outcomes for patients with advanced HCC. Immunotherapies, such as atezolizumab plus bevacizumab and durvalumab plus tremelimumab, are now recommended as first‐line treatments for advanced HCC by international guidelines [2, 3]. In patients for whom combination immunotherapy is unsuitable, lenvatinib serves as an alternative first‐line option. Several real‐world studies have reported comparable outcomes for lenvatinib and atezolizumab‐bevacizumab in first‐line treatment [4, 5, 6, 7]. Given the significant cost differences, lenvatinib is often preferred in clinical practice, particularly in settings where financial constraints or health insurance coverage play a determining role.

Emerging evidence has demonstrated the crucial role of nutritional status in inflammation, immune function, and treatment responses in patients with cancer. Malnutrition is a common concern, and previous studies have shown that preoperative nutritional status is closely linked to prognosis [8, 9]. The geriatric nutritional risk index (GNRI) was developed to assess nutrition‐related risks, particularly in elderly populations [10]; this index incorporates serum albumin levels and the ratio of current to usual body weight, adjusted for sex and height—factors that are easily obtained before treatment. The GNRI has demonstrated prognostic value in various malignancies, including HCC [11, 12, 13]. Similarly, the prognostic nutritional index (PNI), which is calculated based on serum albumin and lymphocyte count, is a widely recognized biomarker for evaluating immuno‐nutritional status and survival outcomes across multiple cancers, including HCC [14, 15]. Previous studies have indicated that the PNI is an effective prognostic marker among patients with early‐stage HCC undergoing various treatment modalities, such as transplantation, hepatic resection, and microwave ablation [16, 17, 18]. However, few studies have comprehensively explored the prognostic ability of the PNI or GNRI in patients with advanced HCC receiving systemic therapies.

Therefore, this retrospective study aimed to evaluate the associations between the PNI and GNRI and the survival outcomes of patients with advanced HCC treated with first‐line lenvatinib in a real‐world setting.

## 2 Methods

### 2.1 Patients

We conducted a retrospective analysis of patients with unresectable HCC who received lenvatinib treatment between June 2020 and December 2023 at Kaohsiung Chang Gung Memorial Hospital. The diagnosis of HCC was established through dynamic imaging modalities, such as computed tomography (CT) and/or magnetic resonance imaging (MRI). If the imaging results were inconclusive, a histological confirmation was obtained via ultrasound‐guided tumor biopsy.

The eligibility criteria were patients with unresectable intermediate or advanced‐stage HCC (based on the Barcelona Clinic Liver Cancer [BCLC] system) with a Child–Pugh classification of A or B who received lenvatinib as a first‐line systemic treatment. Patients were excluded if they had undergone prior systemic therapy, had concurrent malignancies, lacked sufficient clinical data for prognostic evaluation, or were classified as Child–Pugh class C.

Lenvatinib was administered orally, with the initial dosage determined based on the manufacturer's recommendations. Patients weighing 60 kg or more received 12 mg per day, while patients under 60 kg were prescribed 8 mg per day. Dose modifications were made as necessary, depending on the patient's condition and clinical assessment. The use of concurrent therapies alongside lenvatinib was allowed.

Clinical information was collected for each patient, including demographic variables (age, sex, and body mass index [BMI]) and nutritional indices (GNRI and PNI). Tumor‐related characteristics, including Child–Pugh class, viral etiology, BCLC stage, extrahepatic metastasis (EHM), microvascular invasion (MVI), and tumor size, were also recorded. In addition, laboratory data, including liver function tests and alpha‐fetoprotein (AFP) levels, were analyzed. Treatment‐related information, including treatment duration, dose modifications, early discontinuation, concurrent treatments, and posttreatment outcomes, was extracted from the electronic medical records. The study protocol received approval from the Research Ethics Committee of Chang Gung Memorial Hospital (IRB No. 202500306B0).

### 2.2 Definitions of GNRI and PNI

Nutritional status was assessed using the GNRI and PNI. GNRI was calculated using [14.89 × serum albumin (g/L)] + [41.7 × (actual body weight/ideal body weight)]. The ideal body weight (IBW) formulas were height (cm) − 100 − (height [cm] − 150)/4 for males and height (cm) − 100 − (height [cm] − 150)/2.5 for females. If the actual body weight exceeded the IBW, the ratio was capped at 1. The PNI was calculated using PNI = [10 × serum albumin (g/dL)] + [0.005 × total lymphocyte count (/mm^3^)].

For group stratification, we applied predefined cut‐off values from previous studies of patients with HCC to ensure comparability with the literature and maintain clinical interpretability. Specifically, according to the cut‐off used by Kanno et al. in patients with HCC undergoing hepatectomy [13], a GNRI < 98 was defined as low and a GNRI ≥ 98, as high. Likewise, based on the cut‐off reported by Chan et al. for patients with resected early‐stage HCC [17], a PNI < 45 was defined as low and a PNI ≥ 45, as high. In addition, patients with both a PNI ≥ 45 and GNRI ≥ 98 were classified as having favorable nutritional status in our combined nutritional analysis.

### 2.3 Assessment of Treatment Response

Treatment response was assessed using radiologic imaging following the modified Response Evaluation Criteria in Solid Tumors (mRECIST) [19]. Radiologic follow‐up examinations were conducted approximately every 3 months during lenvatinib treatment, or sooner if clinical deterioration was noted. The objective response rate (ORR) was defined as the percentage of patients who achieved either a complete response (CR) or a partial response (PR). The disease control rate (DCR) was defined as the proportion of patients who achieved CR, PR, or stable disease (SD). Progressive disease (PD) was defined as radiologically evident tumor progression at the time of response evaluation.

### 2.4 Assessment of Adverse Events

Lenvatinib was discontinued in cases of severe or intolerable treatment‐related adverse events (TRAEs) or when clinical tumor progression was observed. Dose reduction or temporary interruption was performed according to the lenvatinib prescribing guidance, particularly in patients who developed Grade ≥ 3 TRAEs or intolerable Grade 2 TRAEs. Severe TRAEs (Grade ≥ 3) required dose reduction or treatment interruption until the event improved to Grade 1 or 2, in accordance with the manufacturer's recommendations. Adverse events were assessed and documented at each follow‐up visit by treating clinicians and specialized nurses. Patients were generally followed monthly, with additional visits arranged as needed according to TRAE severity.

### 2.5 Statistical Analysis

Patients were followed up until their final visit, death, or December 2024. Mortality data were collected through follow‐up visits and medical records to ensure accuracy and completeness. Continuous variables are reported as mean ± standard deviation or median with interquartile range, and categorical variables are presented as frequencies and percentages. Group differences were analyzed using the Student's *t*‐test, Mann–Whitney U‐test, chi‐square test, or Fisher's exact test, as appropriate. Overall survival (OS) and progression‐free survival (PFS) were estimated using the Kaplan–Meier method and log‐rank test. Cox proportional hazards regression models were applied for univariate and multivariate analyses. In sensitivity and comparative analyses, albumin‐only Cox models were constructed and model fit/discrimination were compared using Akaike information criterion (AIC) and Harrell's concordance index (C‐index). A *p* < 0.05 in a two‐tailed test was considered statistically significant, and all analyses were performed using SPSS 26 software.

## 3 Results

### 3.1 Baseline Characteristics

**FIGURE 1:**
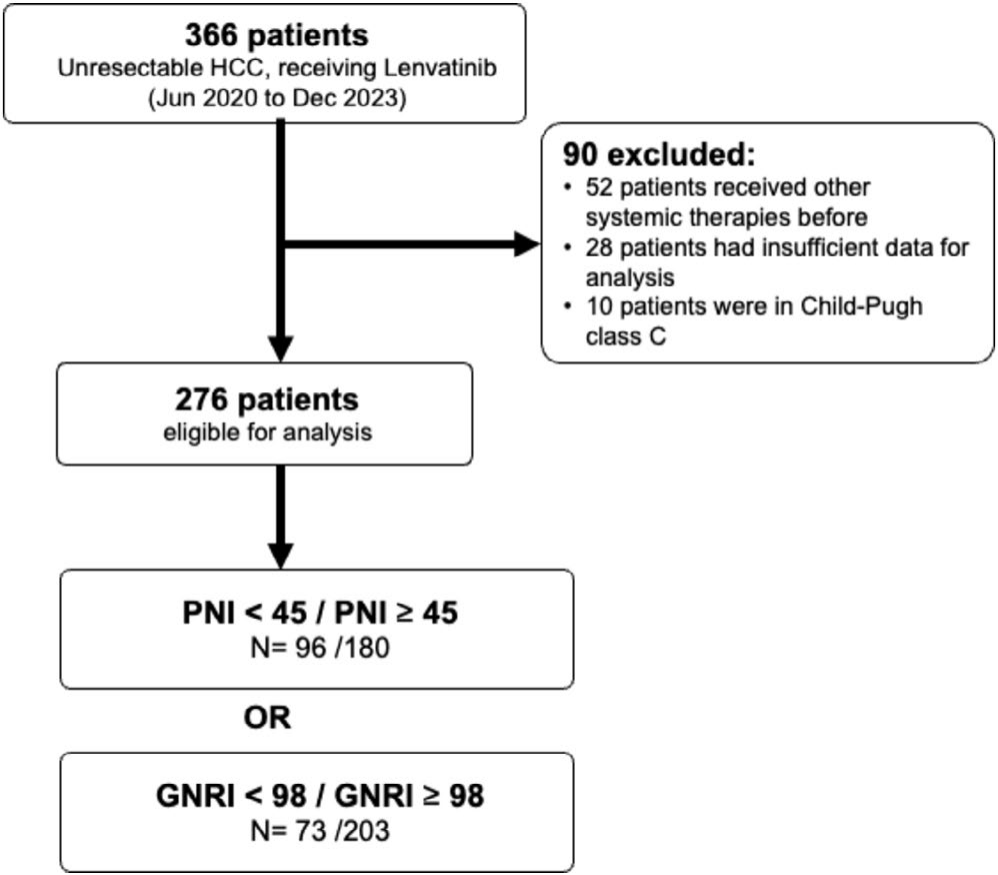
Flowchart of enrolled patients.

The enrollment flowchart for this study is presented in Figure 1. A total of 366 patients with unresectable HCC received lenvatinib at our center between June 2020 and December 2023. Of these, 52 patients were excluded from this study due to prior systemic therapy, 28 patients lacked sufficient clinical data for assessment, and 10 patients had a Child–Pugh class C classification. Consequently, 276 patients were eligible for analysis, of whom 96 patients were classified into the low PNI group and 180 into the high PNI group. Similarly, 73 patients were categorized into the low GNRI group and 203 into the high GNRI group.

Table 1 summarizes the baseline characteristics of all enrolled patients based on the PNI and GNRI classifications. The high PNI group had a significantly higher proportion of patients with Child–Pugh class A compared to the low PNI group (98.3% vs. 85.4%, *p* < 0.001), whereas the low PNI group had a higher proportion of patients with Child–Pugh Class B (14.6% vs. 1.7%, *p* < 0.001); a similar trend was observed for the high GNRI and low GNRI groups. Additionally, the high GNRI group had a significantly higher BMI compared to the low GNRI group (25.8 ± 3.8 vs. 21.9 ± 2.8, *p* < 0.001). Furthermore, concurrent treatment during lenvatinib therapy was more frequent in the high PNI group than in the low PNI group (45.0% vs. 31.3%, *p* = 0.026), whereas this difference was not statistically significant between the GNRI groups (41.9% vs. 35.6%, *p* = 0.350).

**TABLE 1:** Baseline characteristics of patients receiving first‐line Len based on the PNI or GNRI.

	PNI			GNRI		
	< 45 (*n* = 96)	≥ 45 (*n* = 180)	*p*	< 98 (*n* = 73)	≥ 98 (*n* = 203)	*p*
Male sex, *n* (%)	71 (74)	140 (77.8)	0.476	58 (79.5)	153 (75.4)	0.481
Age (years), mean ± SD	66.8 ± 10.9	64.9 ± 11.2	0.174	66.6 ± 11.6	65.2 ± 10.9	0.382
Child–Pugh class						< 0.001
A, *n* (%)	82 (85.4)	177 (98.3)	< 0.001	61 (83.6)	198 (97.5)	
B, *n* (%)	14 (14.6)	3 (1.7)		12 (16.4)	5 (2.5)	
Viral etiology, *n* (%)	69 (71.9)	140 (77.8)	0.276	51 (69.9)	158 (77.8)	0.173
HBV infection, *n* (%)	45 (46.9)	101 (56.1)	0.143	35 (47.9)	111 (54.7)	0.323
HCV infection, *n* (%)	26 (27.1)	41 (22.8)	0.427	18 (24.7)	49 (24.1)	0.929
BCLC stage						0.279
B, *n* (%)	22 (22.9)	34 (18.9)	0.428	18 (24.7)	38 (18.7)	
C, *n* (%)	74 (77.1)	146 (81.1)		55 (75.3)	165 (81.3)	
EHM, *n* (%)	36 (37.5)	83 (46.1)	0.169	28 (38.4)	91 (44.8)	0.338
MVI, *n* (%)	47 (49)	76 (42.2)	0.284	35 (47.9)	88 (43.3)	0.498
Tumor size ≥ 5 cm, *n* (%)	44 (45.8)	72 (40.2)	0.369	34 (47.9)	19 (48.7)	0.934
Tumor number ≥ 3, *n* (%)	64 (66.7)	97 (54.5)	0.262	37 (50.7)	79 (39.1)	0.086
BMI (kg/m^2^), mean ± SD	25.1 ± 4.5	24.6 ± 3.6	0.315	21.9 ± 2.8	25.8 ± 3.8	< 0.001
AST (IU/L), mean ± SD	69.5 ± 42.9	62.5 ± 63.7	0.289	70.9 ± 36.9	62.8 ± 63.1	0.202
ALT (IU/L), mean ± SD	52.2 ± 36.2	53.3 ± 72.2	0.871	55.6 ± 36.2	51.9 ± 69.1	0.569
Bilirubin (IU/L) mean ± SD	1.4 ± 1.0	0.9 ± 0.5	< 0.001	1.3 ± 1.1	1.0 ± 0.6	0.011
Creatine (mg/dL), mean ± SD	1.2 ± 0.9	1.1 ± 0.8	0.346	1.2 ± 1.1	1.1 ± 0.9	0.455
Platelet count (×10^9^/L), mean ± SD	171.2 ± 105	195.2 ± 101	0.007	216.1 ± 130	176 ± 89	0.009
AFP (ng/mL), median (IQR)	181.9 (10.1–1842.2)	50.1 (7.6–754)	0.078	88.3 (9.85–1877.3)	65.9 (8.8–955.3)	0.472
AFP ≥ 400, *n* (%)	36 (37.5)	50 (27.9)	0.103	28 (38.4)	58 (28.7)	0.128
Concurrent treatment, *n* (%)	30 (31.3)	81 (45)	0.026	26 (35.6)	85 (41.9)	0.350
RTO/PBT	6/7	25/11		7/3	24/15	
Nivolumab/pembrolizumab	1/8	8/13		1/7	8/14	
TACE	5	17		7	15	
Treatment stop, *n* (%)	92 (95.8)	170 (94.4)	0.616	71 (97.3)	191 (94.1)	0.290
Treatment duration, months	5.4 ± 4.4	8.3 ± 5.7	< 0.001	5.1 ± 4.0	8.1 ± 5.7	< 0.001
Posttreatment, *n* (%)	30 (33)	98 (58.7)	< 0.001	22 (31.4)	106 (56.4)	< 0.001
Chemotherapy	4	24		5	23	
Ate‐Bev	5	14		2	17	
Nivolumab‐based treatment	4	14		4	14	

Abbreviations: AFP, alpha‐fetoprotein; ALT, alanine aminotransferase; AST, aspartate transaminase; Ate‐Bev, atezolizumab plus bevacizumab; BCLC stage, Barcelona Clinic Liver Cancer stage; BMI, body mass index; EHM, extrahepatic metastasis; GNRI, geriatric nutrition risk index; PBT, proton beam therapy; PNI, prognostic nutrition index; RTO, radiotherapy; TACE, transcatheter arterial chemoembolization.

To better characterize the concomitant treatments, we further summarized the most common treatment categories (Table 1). The major concurrent modalities included radiotherapy/proton beam therapy (RTO/PBT), single‐agent immune checkpoint inhibitor therapy (nivolumab or pembrolizumab), and TACE.

In terms of subsequent management after the failure of first‐line lenvatinib, posttreatment was more frequently administered to patients with better nutritional status. Specifically, posttreatment was provided to 30 patients (33.0%) in the low PNI group compared to 98 patients (58.7%) in the high PNI group (*p* < 0.001), and to 22 patients (31.4%) in the low GNRI group compared to 106 patients (56.4%) in the high GNRI group (*p* < 0.001). The most common subsequent treatment modalities were chemotherapy, atezolizumab plus bevacizumab, and nivolumab‐based treatment.

### 3.2 Treatment Response Based on PNI or GNRI

Treatment response was assessed through radiological imaging. The high PNI group had a CR rate of 6.7%, PR rate of 16.0%, SD rate of 50.9%, and PD rate of 26.4%, and the high GNRI group had a CR rate of 6.8%, PR rate of 15.3%, SD rate of 50.8%, and PD rate of 27.1% (Table 2). Overall, the ORR was significantly higher in the high PNI group compared to the low PNI group (22.7% vs. 11.2%; *p* = 0.041), while the difference in ORR between the high‐ and low GNRI groups was borderline significant (22.1% vs. 10.5%; *p* = 0.055). No significant differences were observed between the PNI and GNRI groups in terms of CR, PR, SD, PD, DCR, or mortality. However, trends suggesting higher ORR in the high PNI and high GNRI groups were observed.

**TABLE 2:** Treatment response of patients receiving lenvatinib based on the PNI or GNRI.

Variables	PNI			GNRI		
	< 45 (*n* = 96)	≥ 45 (*n* = 180)	*p*	< 98 (*n* = 73)	≥ 98 (*n* = 203)	*p*
Treatment response evaluation, *n* (%)^a^	71 (73.9)	163 (90.6)		57 (78.1)	177 (87.2)	
Complete response, *n* (%)	3 (4.2)	11 (6.7)	0.360	2 (3.5)	12 (6.8)	0.222
Partial response, *n* (%)	5 (7)	26 (16)		4 (7.0)	27 (15.3)	
Stable disease, *n* (%)	37 (52.1)	83 (50.9)		30 (52.6)	90 (50.8)	
Progression disease, *n* (%)	26 (36.6)	43 (26.4)		21 (36.8)	48 (27.1)	
Objective response rate	11.2%	22.7%	0.041	10.5%	22.1%	0.055
Disease control rate	63.4%	73.6%	0.114	63.2%	72.9%	0.161
Death, *n* (%)	56 (58.3)	106 (58.9)	0.929	49 (67.1)	113 (55.7)	0.088

Abbreviations: GNRI, geriatric nutrition risk index; PNI, prognostic nutrition index.

^a^ Treatment response based on those who received image evaluation including computer tomography or magnetic resonance image.

### 3.3 TRAEs

TRAEs were assessed for the patients with available medical records, with 210 patients (76%) experiencing adverse events (Table 3). The most frequently reported TRAE was hand‐foot skin reaction, which occurred in 32% of the low PNI group and 37% of the high PNI group. Similarly, 44% of the low GNRI group and 38% of the high GNRI group experienced this adverse event. The second most common TRAE was fatigue, which was reported in 17.8% of the high PNI group and 18.2% of the high GNRI group. Overall, patients in the low PNI or low GNRI groups exhibited a significantly higher incidence of severe TRAEs (17.2% and 17.8%, respectively) compared to the high PNI and high GNRI groups (8.6%, *p* = 0.035 and 9.2%, *p* = 0.05, respectively). However, the total incidence of TRAEs did not differ significantly between groups.

**TABLE 3:** Treatment related adverse events of patients receiving lenvatinib based on PNI or GNRI.

Variables	PNI				GNRI			
	< 45 (*n* = 96)		≥ 45 (*n* = 180)		< 98 (*n* = 73)		≥ 98 (*n* = 203)	
	Any, *n* (%)	Grade ≥ 3, *n* (%)	Any, *n* (%)	Grade ≥ 3, *n* (%)	Any, *n* (%)	Grade ≥ 3, *n* (%)	Any, *n* (%)	Grade ≥ 3, *n* (%)
Total TRAE	65 (69.9)	16 (17.2)^a^	114 (65.1)	15 (8.6)^a^	50 (68.5)	13 (17.8)^a^	129 (66.2)	18 (9.2)^a^
HFSR, *n* (%)	22 (22.9)	4 (4.2)	44 (24.4)	4 (2.3)	15 (20.5)	3 (4.1)	51 (25.1)	5 (2.5)
Fatigue, *n* (%)	20 (20.8)	3 (3.1)	32 (17.8)	5 (2.8)	15 (20.5)	3 (4.1)	37 (18.2)	5 (2.5)
Diarrhea, *n* (%)	17 (17.7)	1 (1.1)	26 (14.4)	1 (0.6)	11 (15.1)	1 (1.4)	32 (15.8)	1 (0.5)
Elevated T‐bil, *n* (%)	14 (14.6)	4 (4.2)	5 (2.8)	2 (1.2)	11 (15.1)	3 (4.1)	8 (3.9)	3 (1.5)
Hypertension, *n* (%)	10 (10.4)	2 (2.1)	19 (10.5)	1 (0.6)	8 (10.9)	1 (1.4)	21 (10.3)	2 (1.0)
Poor appetite, *n* (%)	10 (10.4)	0	15 (8.3)	0	10 (13.6)	0	15 (7.4)	0
Proteinuria, *n* (%)	8 (8.6)	0	8 (4.4)	0	5 (6.8)	0	11 (5.4)	0
Hepatitis, *n* (%)	6 (6.3)	1 (1.1)	10 (5.6)	0	7 (9.6)	0	9 (4.4)	1 (0.5)
Dysphonia, *n* (%)	5 (5.2)	0	8 (4.4)	0	4 (5.5)	0	9 (4.4)	0
Hepatic encephalopathy, *n* (%)	3 (3.1)	1 (1.1)	3 (1.6)	1 (0.6)	4 (5.5)	2 (1.4)	2 (0.9)	0
UGI bleeding, *n* (%)	1 (1.1)	0	4 (2.2)	1 (0.6)	2 (2.7)	0	3 (1.5)	1 (0.5)

Abbreviations: GNRI, geriatric nutrition risk index; HFSR, hand foot skin reaction; PNI, prognostic nutrition index; TRAE, treatment related adverse event; UGI bleeding, upper gastrointestinal bleeding.

^a^ The comparison of any severe TRAE was 0.035 between PNI groups and 0.051 between GNRI groups, respectively.

### 3.4 Associations of PNI and GNRI With PFS and OS

**FIGURE 2:**
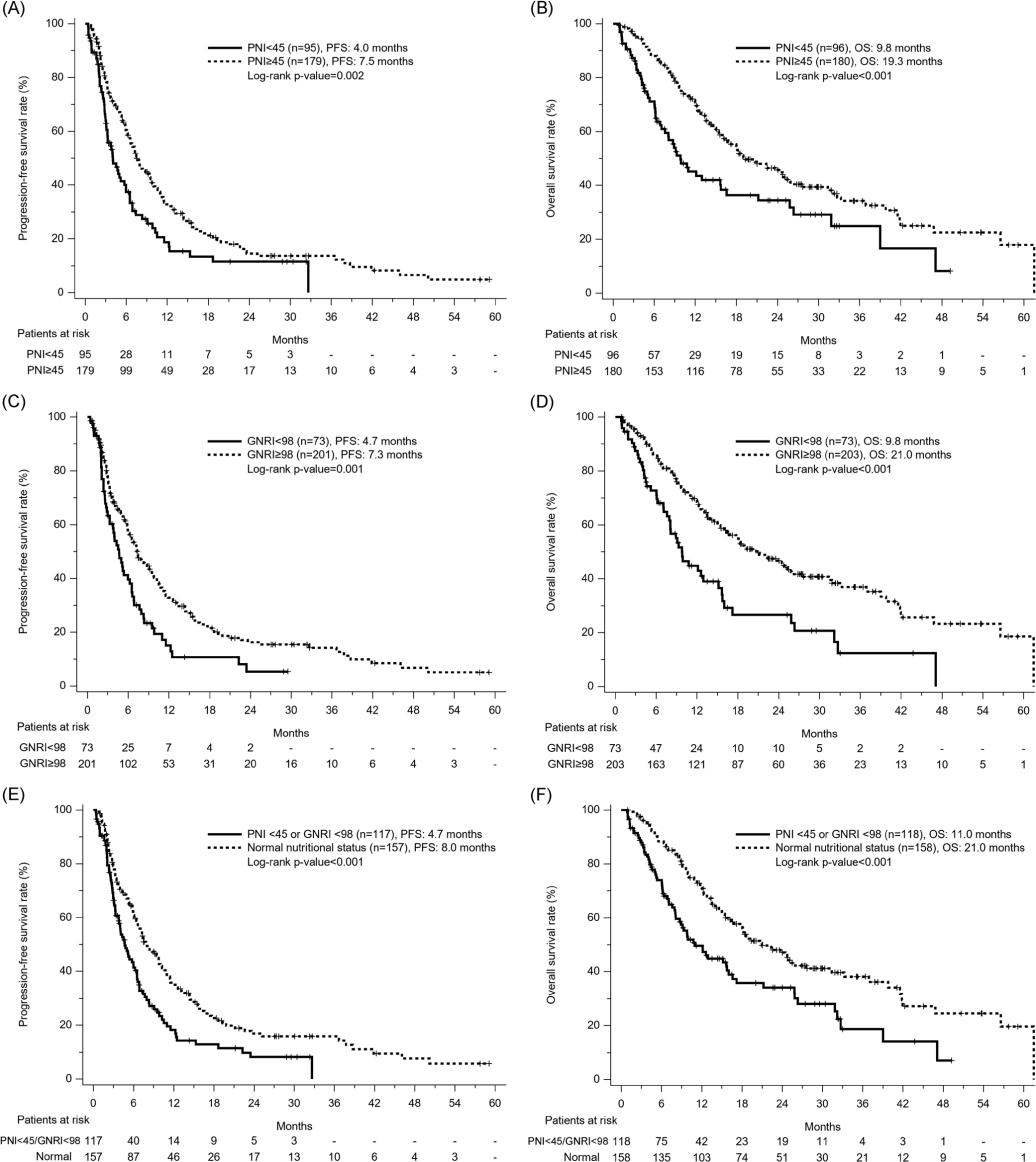
Kaplan–Meier curves of (A) progression‐free survival (PFS) and (B) overall survival (OS) of patients based on the PNI; (C) PFS and (D) OS of patients based on the GNRI; and (E) PFS and (F) OS of patients based on the PNI and GNRI.

Patients in the low PNI group achieved median PFS of 4.0 months, whereas patients in the high PNI group had significantly longer PFS of 7.5 months (*p* = 0.002; Figure 2A). OS was also significantly lower in the low PNI group, which achieved median survival of 9.8 months, compared to 19.3 months in the high PNI group (*p* < 0.001; Figure 2B). Similarly, the low GNRI group had significantly poorer PFS compared to the high GNRI group (4.7 vs. 7.3 months, *p* = 0.001; Figure 2C). The low GNRI group also achieved significantly poorer OS than the high GNRI group (9.8 vs. 21 months, *p* < 0.001; Figure 2D).

Notably, patients with normal nutritional status, that is, both high PNI and high GNRI, achieved significantly better PFS (8.0 vs. 4.7 months, *p* < 0.001; Figure 2E) and OS (21 vs. 11 months, *p* < 0.001; Figure 2F) compared to patients with both low PNI or low GNRI.

### 3.5 Factors Associated With PFS

Three multivariable Cox regression models were constructed to evaluate the prognostic significance of nutritional status for PFS, with PNI ≥ 45, GNRI ≥ 98, and the combined variable (PNI ≥ 45 or GNRI ≥ 98) entered separately (Table 4). In univariate analysis, Child–Pugh Class B (HR: 2.138, *p* = 0.005), MVI (HR: 1.671, *p* < 0.001), main tumor size ≥ 5 cm (HR: 1.398, *p* = 0.016), and AFP ≥ 400 ng/mL (HR: 1.442, *p* = 0.014) were associated with shorter PFS, whereas concurrent treatment (HR: 0.649, *p* = 0.002) was associated with longer PFS. The nutritional variables PNI ≥ 45 (HR: 0.631, *p* = 0.002), GNRI ≥ 98 (HR: 0.604, *p* = 0.001), and the combined variable (PNI ≥ 45 or GNRI ≥ 98) (HR: 0.614, *p* < 0.001) were also significant in univariate analysis.

**TABLE 4:** Factors associated with progression free survival for patients with first‐line lenvatinib.

Variables	Univariate			Multivariate Model 1		
	HR	95% CI	*p*	HR	95% CI	*p*
Age, per year increase	1.004	0.991–1.016	0.579			
Male sex	0.978	0.710–1.347	0.893			
Child–Pugh Class B	2.138	1.260–3.629	0.005			
HBV infection	1.017	0.776–1.333	0.905			
HCV infection	1.105	0.796–1.534	0.551			
EHM	0.955	0.728–1.252	0.739			
MVI	1.671	1.273–2.192	< 0.001	1.569	1.191–2.068	0.001
Main tumor ≥ 5 cm	1.398	1.064–1.836	0.016			
Tumor number ≥ 3	1.275	0.966–1.683	0.086			
AFP ≥ 400 ng/mL	1.442	1.078–1.929	0.014	1.402	1.045–1.882	0.024
Concurrent treatment	0.649	0.493–0.856	0.002	0.709	0.535–0.939	0.016
PNI ≥ 45	0.631	0.471–0.844	0.002	0.677	0.504–0.911	0.010
GNRI ≥ 98	0.604	0.444–822	0.001			
PNI ≥ 45 or GNRI ≥ 98	0.614	0.465–0.812	< 0.001			

Variables	Multivariate Model 2			Multivariate Model 3		
	HR	95% CI	*p*	HR	95% CI	*p*
Age, per year increase						
Male sex						
Child–Pugh Class B						
HBV infection						
HCV infection						
EHM						
MVI	1.530	1.161–2.016	0.003	2.031	1.171–2.016	0.002
Main tumor ≥ 5 cm						
Tumor number ≥ 3						
AFP ≥ 400 ng/mL	1.392	1.037–1.867	0.028	1.415	1.054–1.898	0.021
Concurrent treatment	0.688	0.521–0.908	0.008	0.700	0.530–0.925	0.012
PNI ≥ 45						
GNRI ≥ 98	0.655	0.480–0.892	0.007			
PNI ≥ 45 or GNRI ≥ 98				0.649	0.497–0.874	0.004

Abbreviations: AFP, alpha‐fetoprotein; BCLC stage, Barcelona Clinic Liver Cancer stage; EHM, extrahepatic metastasis; GNRI, geriatric nutrition risk index; PNI, prognostic nutrition index.

In multivariable analysis, MVI, AFP ≥ 400 ng/mL, and concurrent treatment remained independently associated with PFS across all three models. Importantly, PNI ≥ 45 remained an independent protective factor in Model 1 (HR: 0.677, 95% CI: 0.504–0.911, *p* = 0.010), GNRI ≥ 98 remained significant in Model 2 (HR: 0.655, 95% CI: 0.480–0.892, *p* = 0.007), and the combined variable (PNI ≥ 45 or GNRI ≥ 98) remained significant in Model 3 (HR: 0.649, 95% CI: 0.497–0.874, *p* = 0.004).

### 3.6 Factors Associated With OS

Three corresponding multivariable Cox regression models were also constructed for OS using the same revised covariate framework (Table 5). In univariate analysis, Child–Pugh Class B (HR: 3.023, *p* < 0.001), MVI (HR: 1.621, *p* = 0.002), main tumor ≥ 5 cm (HR: 1.799, *p* < 0.001), and AFP ≥ 400 ng/mL (HR: 1.867, *p* < 0.001) were associated with poorer OS. In contrast, concurrent treatment (HR: 0.620, *p* = 0.004) and posttreatment (HR: 0.440, *p* < 0.001) were associated with longer OS. The nutritional variables PNI ≥ 45 (HR: 0.571, *p* < 0.001), GNRI ≥ 98 (HR: 0.497, *p* < 0.001), and combined PNI ≥ 45 or GNRI ≥ 98 (HR: 0.559, *p* < 0.001) were also significantly associated with OS in univariate analysis.

**TABLE 5:** Factors associated with overall survival for patients with first‐line lenvatinib.

Variables	Univariate			Multivariate Model 1		
	HR	95% CI	*p*	HR	95% CI	*p*
Age, per year increase	1.009	0.994–1.024	0.225			
Sex	0.920	0.643–1.315	0.647			
Child–Pugh class	3.023	1.734–5.268	< 0.001			
HBV infection	0.798	0.586–1.088	0.154			
HCV infection	1.070	0.746–1.536	0.713			
EHM	1.0865	0.796–1.480	0.606			
MVI	1.621	1.188–2.211	0.002			
Main tumor ≥ 5 cm	1.799	1.315–2.459	< 0.001	1.789	1.302–2.457	< 0.001
Tumor number ≥ 3	1.236	0.900–1.698	0.191			
AFP ≥ 400 ng/mL	1.867	1.345–2.591	< 0.001	1.664	1.194–2.318	0.003
Concurrent treatment	0.620	0.449–0.858	0.004	0.644	0.462–0.897	0.009
Posttreatment	0.440	0.320–0.606	< 0.001	0.449	0.323–0.626	< 0.001
PNI ≥ 45	0.571	0.471–0.793	< 0.001	0.678	0.491–0.936	0.018
GNRI ≥ 98	0.497	0.353–0.698	< 0.001			
PNI ≥ 45 or GNRI ≥ 98	0.559	0.408–0.766	< 0.001			

Variables	Multivariate Model 2			Multivariate Model 3		
	HR	95% CI	*p*	HR	95% CI	*p*
Age, per year increase						
Sex						
Child–Pugh class						
HBV infection						
HCV infection						
EHM						
MVI	1.510	1.098–2.077	0.011	1.506	1.096–2.069	0.011
Main tumor ≥ 5 cm	1.737	1.263–2.389	< 0.001	1.755	1.278–2.411	< 0.001
Tumor number ≥ 3						
AFP ≥ 400 ng/mL	1.682	1.209–2.342	0.002	1.689	1.213–2.352	0.002
Concurrent treatment	0.643	0.461–0.895	0.009	0.640	0.460–0.892	0.008
Posttreatment	0.465	0.333–0.650	< 0.001	0.455	0.327–0.635	< 0.001
PNI ≥ 45						
GNRI ≥ 98	0.679	0.477–0.964	0.031			
PNI ≥ 45 or GNRI ≥ 98				0.655	0.468–0.915	0.013

Abbreviations: AFP, alpha‐fetoprotein; BCLC stage, Barcelona Clinic Liver Cancer stage; EHM, extrahepatic metastasis; GNRI, geriatric nutrition risk index; PNI, prognostic nutrition index.

In multivariable analysis for OS, main tumor ≥ 5 cm, AFP ≥ 400 ng/mL, concurrent treatment, and posttreatment remained independent prognostic factors across the three models, and MVI remained significant in Models 2 and 3. Importantly, PNI ≥ 45 remained independently associated with longer OS in Model 1 (HR: 0.678, 95% CI: 0.491–0.936, *p* = 0.018), GNRI ≥ 98 remained significant in Model 2 (HR: 0.679, 95% CI: 0.477–0.964, *p* = 0.031), and combined PNI ≥ 45 and GNRI ≥ 98 remained significant in Model 3 (HR: 0.655, 95% CI: 0.468–0.915, *p* = 0.013). In additional sensitivity analyses, serum albumin alone was also significantly associated with PFS and OS. Comparative model performance across albumin‐, PNI‐, and GNRI‐based models were comparable in terms of model fit and discrimination, as summarized in Tables S1A,B and S2.

## 4 Discussion

The current study provides crucial insights into the prognostic value of nutritional indices, particularly the PNI and GNRI, in patients with advanced HCC receiving first‐line lenvatinib treatment. Chan et al. previously demonstrated that the PNI was a significant prognostic factor for OS and disease‐free survival in patients with very early‐ or early‐stage HCC who underwent curative surgery [17]. Moreover, Ho et al. reported that the PNI was the most significant prognostic factor for OS among patients with HCC and could accurately stratify patients into different prognostic groups [20]. Persano et al. further demonstrated that the PNI was an independent prognostic factor for OS and PFS in patients with HCC undergoing first‐line treatment with atezolizumab plus bevacizumab [21]. Lenvatinib is widely administered to patients with unresectable HCC, particularly when immunotherapy is not feasible. This study establishes that the PNI is also a significant prognostic factor for OS and PFS in this group of patients. A PNI cut‐off of 45 was associated with tumor recurrence and OS, with patients in the high PNI group achieving superior response rates (22.7% vs. 11.2%, *p* = 0.004) and longer OS (19.3 vs. 9.8 months, *p* < 0.001) and PFS (7.5 vs. 4.0 months, *p* = 0.002) compared to the low PNI group.

Similarly, GNRI, which includes serum albumin as a variable, reflects both nutritional and inflammatory status. Li et al. discovered that preoperative GNRI could predict severe postoperative complications, and a lower GNRI was linked to poorer OS after hepatectomy in elderly patients with HCC [22]. More recently, Hiraoka et al. found that the GNRI is an effective nutritional prognostic tool for complications related to muscle volume loss in patients with HCC treated with atezolizumab plus bevacizumab [23]. Our study also confirms that the GNRI has prognostic significance in patients receiving lenvatinib, with the high GNRI (cut‐off: 98) group exhibiting longer PFS (7.3 vs. 4.7 months, *p* = 0.001) and OS (21 vs. 9.8 months, *p* < 0.001) compared to the low GNRI group.

Although both the PNI and GNRI have been separately validated as prognostic markers of outcomes in HCC, there is limited research on their combined use in HCC. Yang et al. confirmed that both indices can predict clinical outcomes and malnutrition postliver resection in patients with HCC [24]. More recently, Tsukagoshi et al. further demonstrated that patients with both low PNI and low GNRI had significantly poorer OS and recurrence‐free survival after hepatic resection [25]. Our current study is the first to explore the association between the combination of GNRI and PNI in patients with advanced HCC receiving systemic lenvatinib. Consistent with the previous studies, our analysis found that patients with both low PNI and GNRI achieved significantly poorer OS (11 vs. 21 months, *p* < 0.001) and PFS (4.7 vs. 8 months, *p* < 0.001) compared to patients with normal nutritional status (i.e., both high PNI and GNRI). Thus, these results suggest that the combined analysis of these indices may enhance prognostic accuracy in patients with HCC receiving lenvatinib.

In real‐world practice, patients receiving lenvatinib often undergo additional locoregional or systemic therapies during treatment, and these concomitant interventions may influence treatment responses and survival. Thus, we detailed the major concurrent treatment categories in Table 1, which included RTO/PBT, single‐agent immune checkpoint inhibitor therapy, and TACE. As concurrent treatment may act as a treatment‐related confounder, we also included this variable in the multivariable models. Notably, although concurrent treatment remained independently associated with survival outcomes, the prognostic significance of PNI/GNRI was preserved after adjustment for concurrent treatment exposure, which supports the robustness of our findings.

To further validate the prognostic significance of the PNI and GNRI, we employed three separate multivariate models to confirm their independent associations with the patient outcomes. In this analysis, PNI < 45, GNRI < 98, and the combination of both low PNI and low GNRI were all independently associated with poorer PFS and OS. The multivariate models consistently indicated that malnutrition, as reflected by these indices, remained significant prognostic markers of patient outcomes—even after adjusting for other clinical variables such as tumor burden and liver function. These findings further demonstrate that nutritional status is a crucial and independent determinant of prognosis in patients with advanced HCC receiving systemic therapy.

Serum albumin is an established prognostic biomarker in advanced HCC and, as expected, remained informative in our cohort. Importantly, the clinical value of PNI and GNRI lies in their role as pragmatic composite indices rather than as substitutes for albumin alone. By integrating albumin with complementary domains of host reserve—immune status (lymphocyte count) in PNI and weight‐related nutritional risk in GNRI—these indices provide a standardized, bedside‐applicable approach for risk stratification and clinical communication. In real‐world practice, a composite score may be more actionable than interpreting individual parameters separately, particularly when clinicians need rapid stratification before treatment initiation. Accordingly, our supplementary analyses support that PNI/GNRI offer clinically useful prognostic assessment while acknowledging albumin as a key contributor to their predictive signal.

With respect to TRAEs, previous studies have reported that multiple factors—such as age, sarcopenia, BMI, weight loss, and liver functional reserve—can affect the tolerability of lenvatinib in patients with HCC. Kinoshita et al. also suggested that the GNRI may be independently associated with the duration of lenvatinib use [26]. In our study, while the total incidence of TRAEs was similar between groups, severe TRAEs were significantly more frequent in the low PNI group and low GNRI group compared to the corresponding high groups (17.2% vs. 8.6% for PNI, *p* = 0.035; 17.8% vs. 9.2% for GNRI, *p* = 0.051). A higher rate of severe TRAEs may lead to earlier treatment discontinuation, as reflected in the shorter treatment duration for the low PNI group (5.4 months) and low GNRI group (5.1 months) compared to the high PNI group (8.3 months, *p* < 0.001) and high GNRI group (8.1 months, *p* < 0.001). This finding suggests that a lower incidence of TRAEs may allow patients with advanced HCC to better tolerate systemic treatment and thus enable the treatment to achieve maximum efficacy. Predicting the tolerability of cancer treatment before initiating therapy is crucial to optimize treatment outcomes. In this respect, the PNI and GNRI are simple, accessible tools that can be easily calculated by clinicians. Furthermore, better nutritional status is associated with more favorable outcomes in patients with cancer. Proper nutrition enhances immune function and supports the ability of the body reserves to withstand the disease burden. Therefore, regular monitoring of a patient's PNI and GNRI may allow clinicians to proactively manage complications or adverse events and potentially increase nutritional support to ensure optimal treatment results.

The current study has several limitations. First, its retrospective design may have introduced selection bias, and prospective studies are needed to validate our findings. Second, rather than deriving cut‐offs for this single‐center cohort, the cut‐off values for the GNRI and PNI (98 and 45, respectively) were adopted from prior studies of HCC to facilitate comparability. Third, the study cohort consisted primarily of Asian patients, which may limit the generalizability of our results. Finally, although the PNI and GNRI are clinically useful markers, they do not fully capture all dimensions of malnutrition, such as loss of muscle mass.

## 5 Conclusion

In conclusion, our study indicated that low PNI and GNRI scores are significant prognostic factors for poorer outcomes in patients with advanced HCC receiving first‐line lenvatinib, as well as TRAEs and posttreatment eligibility. Thus, these nutritional indices—either alone or in combination—may have clinical relevance as simple and useful tools that enable clinicians to identify patients who may not benefit from lenvatinib and adjust treatment plans accordingly.

## Ethics Statement

This study was approved by the Institutional Review Board of Chang Gung Memorial Hospital (IRB No. 202500306B0) and conducted in accordance with the Declaration of Helsinki. The IRB also approved a waiver of additional written consent for retrospective data collection.

## Conflicts of Interest

The authors declare no conflicts of interest.

## Supporting information


**Table S1:** (A) Univariate and multivariable Cox regression analyses evaluating serum albumin for progression‐free survival (albumin‐only model). (B) Univariate and multivariable Cox regression analyses evaluating serum albumin for overall survival (albumin‐only model).
**Table S2:** Model performance comparison between albumin‐, PNI‐, and GNRI‐based models.

## Data Availability

The data that support the findings of this study are available from the corresponding author upon reasonable request.
