# Supplementary material for: Predictive Value of Nutrition Indices in Advanced Hepatocellular Carcinoma Patients Treated With Lenvatinib
Source: Kaohsiung J Med Sci. 2026 May 7:e70230. Online ahead of print. doi: 10.1002/kjm2.70230 (PMC13399649; doi:10.1002/kjm2.70230)
Supplement: Supplementary file 1 — Table S1: (A) Univariate and multivariable Cox regression analyses evaluating serum albumin for progression‐free survival (albumin‐only model). (B) Univariate and multivariable Cox regression analyses evaluating serum albumin for overall survival (albumin‐only model). Table S2: Model performance comparison between albumin‐, PNI‐, and GNRI‐based models. [file KJM2-9999-e70230-s001.docx]

**Supplementary Table S1A. Univariate and multivariable Cox regression analyses evaluating serum albumin for Progression-Free Survival (albumin-only model).**

|  | Univariate | | | Multivariate Model 1 | | |
| --- | --- | --- | --- | --- | --- | --- |
| Variable | H.R. | 95% CI | p-value | H.R. | 95% CI | p-value |
| Age, per year increase | 1.004 | 0.991-1.016 | 0.579 |  |  |  |
| Male sex | 0.978 | 0.710-1.347 | 0.893 |  |  |  |
| Child-Pugh class B | 2.138 | 1.260-3.629 | 0.005 | 2.206 | 1.277-3.812 | 0.005 |
| HBV infection | 1.017 | 0.776-1.333 | 0.905 |  |  |  |
| HCV infection | 1.105 | 0.796-1.534 | 0.551 |  |  |  |
| EHM | 0.955 | 0.728-1.252 | 0.739 | 1.376 | 1.01-1.974 | 0.043 |
| MVI | 1.671 | 1.273-2.192 | <0.001 | 1.853 | 1.368-2.510 | <0.001 |
| Main tumor ≥ 5cm | 1.398 | 1.064-1.836 | 0.016 |  |  |  |
| Tumor number ≥ 3 | 1.275 | 0.966-1.683 | 0.086 |  |  |  |
| AFP ≥400ng/ml | 1.442 | 1.078-1.929 | 0.014 |  |  |  |
| Concurrent treatment | 0.649 | 0.493-0.856 | 0.002 | 0.678 | 0.513-0.897 | 0.007 |
| Albumin, per unit increase | 0.659 | 0.502-0.863 | 0.002 |  |  |  |

Abbreviations: AFP, alpha-fetoprotein; BCLC stage, Barcelona Clinic Liver Cancer stage; EHM, extra-hepatic metastasis.

**Supplementary Table S1B. Univariate and multivariable Cox regression analyses evaluating serum albumin for Overall Survival (albumin-only model).**

|  | Univariate | | | Multivariate Model 1 | | |
| --- | --- | --- | --- | --- | --- | --- |
| Variable | H.R. | 95% CI | p-value | H.R. | 95% CI | p-value |
| Age, per year increase | 1.009 | 0.994-1.024 | 0.225 |  |  |  |
| Sex | 0.920 | 0.643-1.315 | 0.647 |  |  |  |
| Child-Pugh class | 3.023 | 1.734-5.268 | <0.001 |  |  |  |
| HBV infection | 0.798 | 0.586-1.088 | 0.154 |  |  |  |
| HCV infection | 1.070 | 0.746-1.536 | 0.713 |  |  |  |
| EHM | 1.086 | 0.796-1.480 | 0.606 |  |  |  |
| MVI | 1.621 | 1.188-2.211 | 0.002 |  |  |  |
| Main tumor ≥ 5cm | 1.799 | 1.315-2.459 | <0.001 | 1.692 | 1.231-2.327 | 0.001 |
| Tumor number ≥ 3 | 1.236 | 0.900-1.698 | 0.191 |  |  |  |
| AFP ≥ 400 ng/ml | 1.867 | 1.345-2.591 | <0.001 | 1.677 | 1.205-2.335 | 0.002 |
| Concurrent treatment | 0.620 | 0.449-0.858 | 0.004 | 0.664 | 0.477-0.925 | 0.016 |
| Post treatment | 0.440 | 0.320-0.606 | <0.001 | 0.475 | 0.340-0.662 | <0.001 |
| Albumin, per unit increase | 0.461 | 0.339-0.627 | 0.001 | 0.684 | 0.442-0.824 | 0.022 |

Abbreviations: AFP, alpha-fetoprotein; BCLC stage, Barcelona Clinic Liver Cancer stage; EHM, extra-hepatic metastasis.

**Supplementary Table S2. Model performance comparison between albumin-, PNI-, and GNRI-based models***

| **Endpoint** | **Model** | **Number** | **AIC** | **C-index** |
| --- | --- | --- | --- | --- |
| PFS | Albumin-model | 276 | 1977.448 | 0.650 |
| PFS | PNI-model | 276 | 1976.961 | 0.653 |
| PFS | GNRI-model | 276 | 1976.594 | 0.648 |
| OS | Albumin-model | 276 | 1456.770 | 0.717 |
| OS | PNI-model | 276 | 1452.054 | 0.723 |
| OS | GNRI-model | 276 | 1458.197 | 0.714 |

Abbreviations: **AIC**, **Akaike Information Criterion; C-index**, **Concordance Index;** GNRI, geriatric nutrition risk index; OS, overall survival; PFS, progression-free survival; PNI, prognostic nutrition index.

* Model specification (PFS): nutritional variable (Albumin or PNI_45 or GNRI_98) + MVI + AFP400 + Concurrent_treatment.

Model specification (OS): nutritional variable (Albumin or PNI_45 or GNRI_98) + MVI + TumorSize5cm + AFP400 + Concurrent_treatment + Post_treatment.

AIC was calculated from the Cox partial likelihood (AIC = −2 log-likelihood + 2k).

C-index represents Harrell’s concordance index based on the Cox linear predictor. All models were fitted on the same dataset (complete cases) to ensure comparability of AIC and C-index.
